# Construct Validation of a Multidimensional Computerized Adaptive Test for Fatigue in Rheumatoid Arthritis

**DOI:** 10.1371/journal.pone.0145008

**Published:** 2015-12-28

**Authors:** Stephanie Nikolaus, Christina Bode, Erik Taal, Harald E. Vonkeman, Cees A. W. Glas, Mart A. F. J. van de Laar

**Affiliations:** 1 Department of Psychology, Health & Technology, Faculty of Behavioral, Management and Social Sciences, University of Twente, Enschede, The Netherlands; 2 Department of Research Methodology, Measurement and Data Analysis, Faculty of Behavioral, Management and Social Sciences, University of Twente, Enschede, The Netherlands; 3 Department of Rheumatology and Clinical Immunology, Medical Spectrum Twente, Enschede, The Netherlands; 4 Expert Center for Chronic Fatigue, Radboud University Medical Center, Nijmegen, The Netherlands; University of Geneva, SWITZERLAND

## Abstract

**Objective:**

Multidimensional computerized adaptive testing enables precise measurements of patient-reported outcomes at an individual level across different dimensions. This study examined the construct validity of a multidimensional computerized adaptive test (CAT) for fatigue in rheumatoid arthritis (RA).

**Methods:**

The ‘CAT Fatigue RA’ was constructed based on a previously calibrated item bank. It contains 196 items and three dimensions: ‘severity’, ‘impact’ and ‘variability’ of fatigue. The CAT was administered to 166 patients with RA. They also completed a traditional, multidimensional fatigue questionnaire (BRAF-MDQ) and the SF-36 in order to examine the CAT’s construct validity. A priori criterion for construct validity was that 75% of the correlations between the CAT dimensions and the subscales of the other questionnaires were as expected. Furthermore, comprehensive use of the item bank, measurement precision and score distribution were investigated.

**Results:**

The a priori criterion for construct validity was supported for two of the three CAT dimensions (severity and impact but not for variability). For severity and impact, 87% of the correlations with the subscales of the well-established questionnaires were as expected but for variability, 53% of the hypothesised relations were found. Eighty-nine percent of the items were selected between one and 137 times for CAT administrations. Measurement precision was excellent for the severity and impact dimensions, with more than 90% of the CAT administrations reaching a standard error below 0.32. The variability dimension showed good measurement precision with 90% of the CAT administrations reaching a standard error below 0.44. No floor- or ceiling-effects were found for the three dimensions.

**Conclusion:**

The CAT Fatigue RA showed good construct validity and excellent measurement precision on the dimensions severity and impact. The dimension variability had less ideal measurement characteristics, pointing to the need to recalibrate the CAT item bank with a two-dimensional model, solely consisting of severity and impact.

## Introduction

Many patients with rheumatoid arthritis (RA) experience fatigue [1, 2] and describe it as a multidimensional experience [3–6] and an annoying symptom with far-reaching consequences for daily life [7, 8]. Nevertheless, most questionnaires for fatigue in RA are unidimensional and were not developed from the patients’ perspective [9, 10]. Patients’ experience is necessary to gain insight into their subjective symptoms and to develop content valid items [11–13]. Our aim was to improve the measurement of fatigue in RA by incorporating the perspective of patients and by using modern psychometrics. To achieve this goal, we developed a multidimensional computerized adaptive test–the ‘CAT Fatigue RA’.

In a computerized adaptive test (CAT), the computer automatically selects items from a large item bank and subsequently selects the next question based on the previous answer entered by the person completing the CAT. In contrast to traditional questionnaires, CAT measurements for fatigue are more precise since the CAT increasingly discriminates and asks questions based on individuals’ answers regarding their level of fatigue [14]. For the computerized selection of the best matching items, an item pool has to be scaled according to item response theory (IRT). With IRT, item parameters can be assessed for each item independently so that it is known which level of fatigue is represented by the items. This information is required to ideally match the items to the patient’s individual level and for inter-individual comparisons even when patients complete different items [14].

We previously published the development of our item pool and its calibration with IRT. To summarize these studies, first, the fatigue experience of patients was investigated [6, 15]. Then, a large item pool was developed. It contained items of existing questionnaires and new items that had been constructed based on our interview material. This item pool was evaluated by patients and professionals in a Delphi study [9, 16, 17] and finally calibrated with multidimensional IRT [18]. Based on the results of the calibration, the multidimensional CAT Fatigue RA was constructed. It contains 196 items in three dimensions: ‘severity’, ‘impact’ and ‘variability’ of fatigue. According to our knowledge, this is the first CAT that has been developed from the perspective of patients with RA. Another novelty is its multidimensionality. Most of the existing CATs are unidimensional or based on two separately calibrated item banks [19–21]. For example within the PROMIS initiative, two fatigue item banks (experience and impact) have been developed for computerized adaptive testing in the general population and different chronic conditions [21]. If factor analysis and theories based on clinical experience clearly point to multidimensionality, multidimensional IRT models should be applied, despite their being far more complex than unidimensional IRT models [22]. The multidimensionality of fatigue in RA was raised by patients [3–6] but also supported by statistical techniques, and the best possible IRT model was three-dimensional [18]. Multidimensional computerized adaptive testing (MCAT) is fairly new [23], but studies have demonstrated that it can lead to precise and efficient measurements of health outcomes [24, 25]. The cross-information provided by items of correlated dimensions facilitates selection of the most informative items, leading to equal or even higher precision with approximately one-third fewer items than would be needed in unidimensional adaptive testing [26].

The objective of this study was to examine the construct validity of our CAT Fatigue RA. Moreover, the comprehensive use of items of the item bank, measurement precision and the distribution of scores were investigated. These aspects provide specific information about interpretability, reliability and validity of the CAT Fatigue RA, which are important measurement properties for the evaluation of health-related patient-reported outcomes (HR-PRO) [27, 28].

## Materials and Methods

### Patients

Consecutive outpatients with RA from five hospitals in The Netherlands were recruited between the beginning of September and the beginning of December 2013 via the web system ROMA (Rheumatology Online Monitor Application). This system is currently used at the Arthritis Centre Twente and other hospitals of the Dutch Rheumatoid Arthritis Monitoring (DREAM) collaboration [29]. Upon logging into the web system, patients with RA were invited to participate. Once they agreed, the patients were automatically led via the website to the questionnaires belonging to the study. We did not apply any exclusion criteria such as the presence of co-morbidity or a certain disease duration since it was intended to include a representative sample of outpatients with RA. The ethical committee of the University of Twente approved the study.

### Measures

#### CAT Fatigue RA

The multidimensional CAT Fatigue RA measures fatigue on three dimensions [18]. The dimension ‘severity’ contains 13 items about severity, duration, and frequency. The dimension ‘impact’ consists of 169 items about the following topics: cognition/concentration, negative emotions/mood, energy, sleep/rest, body feeling, coping and consequences. The dimension ‘variability’ has 14 items about changes in fatigue and perceived causes. The level of fatigue is expressed in theta values. Theta values are the usual unity in IRT and CAT for the estimation of the construct under consideration, and values are expressed on a metric with a mean of zero and a standard deviation of 1 [22]. Higher thetas indicate higher fatigue levels.

The basis for the construction of the CAT Fatigue RA was the previously calibrated item bank [18]. A between-items multidimensional IRT model was applied, whereby it was assumed that the item bank pertained to a limited number of correlated latent dimensions and that every item loaded in one dimension only. The algorithm of the CAT was based on research on multidimensional adaptive testing by Segall [26]. Therefore, item selection follows Bayesian principles; that is, the item that has the greatest potential to reduce the statistical uncertainty about the fatigue level of the patient is selected from the potential items in the bank [26].

The CAT algorithm was implemented in ROMA. To determine start- and stopping-rules, simulations of the CAT administration were conducted with about 1000 virtual patients. The optimal measurement precision on the three fatigue dimensions was reached with the following characteristics: two random start items per dimension, at least five items per dimension, and a total number of 20 administered items in the CAT. To ensure that the transformation of the CAT algorithm to ROMA was correct, we conducted several test calculations and checked whether both algorithms provided the same fatigue estimates. The criterion of 100% conformity was reached.

Based on the results of a usability test [30], a brief text was included before the start of the CAT to inform patients that some items might appear similar. Moreover, the response option ‘not applicable’ was added to six items of the impact dimension (e.g. items about the impact of fatigue on work). If a patient chooses this option, the CAT selects, as a substitute, the second optimal item for that particular patient at that point in the test.

#### BRAF-MDQ and BRAF-NRS

The Bristol Rheumatoid Arthritis Fatigue Multi-Dimensional Questionnaire (BRAF-MDQ) was developed in the UK [10] and officially translated into Dutch [31]. The BRAF-MDQ contains 20 items and measures four dimensions: physical fatigue, living with fatigue, cognition and emotion. Sum scores can be calculated for each dimension separately. Besides the BRAF-MDQ, patients in our study also filled in the three Bristol Rheumatoid Arthritis Fatigue Numerical Rating Scales (BRAF-NRSs). These eleven-point NRSs measure fatigue severity, effect and coping. Higher scores on the NRS about fatigue severity and effect have a negative meaning while a higher score on the NRS about coping with fatigue indicates a positive outcome (better coping). The BRAF-MDQ showed good internal consistency and, along with the BRAF-NRSs, good criterion validity, construct validity and sensitivity to change [10, 32]. Only the BRAF-NRS coping was not sensitive to change in a pharmacological intervention, suggesting that coping with fatigue is a concept different from severity and effect and should be assessed separately [32].

#### MOS 36-item short-form health survey (SF-36)

The SF-36 [33] contains eight subscales: physical functioning, role limitations because of physical health problems, bodily pain, social functioning, mental health, role limitations because of emotional problems, vitality, and general health perceptions. Standardized scores from 0–100 were calculated, whereby lower scores indicate poorer health- related quality of life (HRQoL). The SF-36 is an adequate instrument to measure health status in Dutch patients with RA [34].

### Analyses

The data used for the following analyses are available as online supporting files.

#### Construct validity

To examine construct validity correlations between the CAT Fatigue RA and the BRAF-MDQ, the BRAF-NRS and SF-36 were calculated.

We expected high correlations (r ≥ .60) between the scores on the dimensions of the CAT and the dimensions of the BRAF-MDQ, the BRAF-NRS scales for severity and effect of fatigue, and the SF-36 subscale vitality (Table 1). All of these dimensions are validated measurements of fatigue. Furthermore, we expected a moderate association between the CAT dimensions and the other subscales of the SF-36 (r > .30 and < .60) and the BRAF-NRS coping. The constructs measured by those SF-36 subscales (e.g. pain, mental health) are closely related to, but different from fatigue [35]. Also, according to Dures et al. [32], the BRAF-NRS coping with fatigue is assumed to measure a concept different from severity and effect.

**Table 1:** Hypothesised strength of correlations with CAT severity, impact and variability.

Expected strength of correlations	
High (r ≥ .60)	BRAF-MDQ physical, living, emotion, cognition, BRAF-NRS severity, effect, SF-36 vitality
Moderate (r > .30 and < .60)	SF-36 physical functioning, role limitations because of physical health problems, bodily pain, social functioning, mental health, general health perceptions, role limitations because of emotional problems, BRAF-NRS coping

As generally recommended for large-scale surveys [36], plausible values were used to estimate the correlations of the other variables with the CAT scores because the latter are estimates with estimation errors rather than directly observed variables. We applied the a priori criterion for construct validity such that at least 75% of the specified hypotheses needed to be supported by the analyses [37].

#### Comprehensive use of items of the item bank

We checked the frequency of use of each item to find out which proportion of items from the item pool had been selected. The dimensions severity and variability contained far less items than the impact dimension, consequently, severity and variability items had a lower chance to be selected than impact items. Moreover, at least five items per dimension were provided to each patient according to the administration rules.

#### Measurement precision

We expected excellent measurement precision (mean SE ≤ 0.32) for the dimensions severity and impact and lower but adequate measurement precision for variability. This hypothesis was based on previous research [38], whereby the standard error (SE) on the dimension severity and impact always reached a level of ≤ 0.32 before the end of the CAT administration of 20 items. This SE is equivalent to a reliability of r = 0.90, which indicates excellent reliability of a CAT [39, 40]. The SE of the dimension variability was larger, but even the largest final SE of this dimension was still equivalent to r = 0.81, reflecting good reliability [39, 12]. The SEs of the fatigue scores (theta values) per dimension were assessed per patient. Subsequently, the mean SE, its standard deviation, minimum and maximum values and values on the percentiles were calculated for each of the three dimensions. The criterion of 0.32 for excellent reliability can be applied to each of the CAT Fatigue RA dimensions separately. This is because the theta-distribution on each dimension has a standard deviation of 1, so the SE of 0.32 entails a proportion of true variance of 0.90, analogous to the unidimensional case.

#### Score distribution

We assumed minimal floor- and ceiling-effects due to the adaptive selection mechanism of a CAT. To examine floor- and ceiling-effects, the distribution of theta scores in the sample were described and graphically displayed. When more than 15% of the CAT administrations led to the highest or lowest possible score, floor- or ceiling-effects were considered present [37].

For the calculation of the correlations needed for the construct validation, a sample size of at least 123 participants is required to detect a significant correlation of .25 with a statistical power of (1-beta) = .80 in a two-tailed test with an alpha of 0.05. This sample size is also adequate for the other analyses.

## Results

In total, 166 patients participated in this study. Their mean scores on the CAT are displayed in Table 2, and their mean scores on the other instruments are displayed in Table 3.

**Table 2:** Theta-scores per CAT dimension.

	CAT severity	CAT impact	CAT variability
Mean theta score	-0.18	-0.22	-0.33
Standard deviation	1.32	1.25	0.88
Minimum	-3.06	-3.50	-2.87
Maximum	2.66	2.50	1.54
Percentiles			
25	-0.94	-0.87	-0.72
50	-0.12	-0.00	-0.19
75	0.81	0.63	0.23
Number (percentage) of scores above 2	8 (4.8%)	2 (1.2%)	0 (0%)
Number (percentage) of scores below 2	18 (10.8%)	18 (10.8%)	13 (7.8%)

Theta scores have a mean of 0 and SD of 1, higher scores indicate higher fatigue.

**Table 3:** Sample means on BRAF-MDQ (N = 164), BRAF-NRS (N = 165) and SF-36 (N = 165).

Measure	Mean (minimum score–maximum score)	Standard deviation
BRAF-MDQ physical	10.59 (0–21)	5.85
BRAF-MDQ living	4.37 (0–21)	4.00
BRAF-MDQ emotion	1.95 (0–9)	2.38
BRAF-MDQ cognition	2.79 (0–15)	2.97
BRAF-NRS severity	4.31 (0–9)	2.54
BRAF-NRS effect	4.02 (0–10)	2.66
BRAF-NRS coping	6.61 (0–10)	2.32
SF-36 Physical Functioning (PF)	65.76 (5–100)	24.54
SF-36 Role Physical (RP)	57.01 (0–100)	26.00
SF-36 Bodily Pain (BP)	61.38 (12–100)	19.24
SF-36 General Health (GH)	54.83 (5–97)	18.90
SF-36 Vitality (VT)	55.95 (0–100)	19.67
SF-36 Social Functioning (SF)	77.80 (0–100)	22.14
SF-36 Role Emotional (RE)	75.10 (0–100)	26.26
SF-36 Mental Health (MH)	75.36 (25–100)	17.07

The sample consisted of 72 men and 94 women, diagnosed with RA and with a mean age of 57.64 years (SD = 10.60; range: 23–83 years) and a mean disease duration of 9.42 years (SD = 10.17; range: 0–69 years).

### Construct validity

In conformity with our hypotheses, high correlations (r ≥ .60) were found between the scores on the severity and impact dimensions of the CAT and all dimensions of the BRAF-MDQ, the BRAF-NRS for severity and effect of fatigue, and the SF-36 subscale vitality (Table 4). The hypotheses regarding the association between those scales and the CAT variability dimension could only be supported for the BRAF physical dimension. All other correlations were lower than expected. However, they were still moderate, ranging between 0.39 (BRAF emotion) and 0.56 (BRAF-NRS severity).

**Table 4:** Correlations CAT with BRAF-MDQ, BRAF-NRS and SF-36.

	CAT severity	CAT impact	CAT variability
BRAF physical	**0.88**	**0.81**	**0.60**
BRAF living	**0.73**	**0.71**	0.54
BRAF emotion	**0.62**	**0.62**	0.39
BRAF cognition	**0.65**	**0.64**	0.45
BRAF-NRS severity	**0.81**	**0.76**	0.56
BRAF-NRS effect	**0.74**	**0.73**	0.54
BRAF-NRS coping	-0.30	**-0.36**	-0.20
SF-36 Physical Functioning (PF)	**-0.53**	**-0.55**	**-0.40**
SF-36 Role Physical (RP)	-0.60	-0.62	**-0.46**
SF-36 Bodily Pain (BP)	**-0.50**	**-0.53**	**-0.39**
SF-36 General Health (GH)	**-0.55**	**-0.51**	**-0.40**
SF-36 Vitality (VT)	**-0.74**	**-0.74**	-0.50
SF-36 Social Functioning (SF)	**-0.58**	-0.61	**-0.42**
SF-36 Role Emotional (RE)	**-0.53**	**-0.56**	**-0.35**
SF-36 Mental Health (MH)	**-0.51**	**-0.54**	**-0.34**
Number of confirmed hypotheses	13 out of 15	13 out of 15	8 out of 15
Percentage confirmed hypotheses	87%	87%	53%

Bold numbers indicate that the correlation is in line with our hypotheses.

As expected, most of the associations between the CAT dimensions and the SF-36 subscales and the BRAF-NRS coping were moderate (r > .30 and < .60). Exceptions were slightly higher correlations between the SF-36 subscale role limitations because of physical health problems and the CAT dimensions severity (.60) and impact (.62), and the SF-36 subscale social functioning and the CAT impact dimension (.61). Moreover, the association between the CAT severity and variability dimensions and the BRAF-NRS coping were slightly lower than expected (0.30 and 0.20).

With 87% of the hypotheses supported (Table 4), the construct validity of the dimensions severity and impact of the CAT Fatigue RA was confirmed. Regarding the dimension variability, 53% of the correlations were as expected.

### Comprehensive use of items of the item bank

Table 5 provides an overview of the frequency of item usage during the 166 CAT administrations.

**Table 5:** Frequency of item usage per CAT dimension in 166 CAT administrations.

	Severity (13 items)	Impact (169 items)	Variability (14 items)
Item not used	-	21 (12.4%)	-
≤ 25 times	1 (7.7%)	131 (77.5%)	4 (28.6%)
26–50 times	4 (30.1%)	7 (4.1%)	5 (35.7%)
51–100 times	6 (46.2%)	8 (4.7%)	2 (14.3%)
101–125 times	2 (15.4%)	2 (1.2%)	-
126–150	-	-	1 (7.1%)
> 150	-	-	2 (14.3%)

Within all the dimensions together, 89.3% of the items were selected. A total of 10.7% of the items remained unused, however, those items all belonged to the largest dimension, namely the impact dimension, which contained about 10 times more items than either the severity or variability dimension.

### Measurement precision

The measurement precision on the three CAT dimensions turned out as expected (Table 6). For the dimensions severity and impact, a mean standard error (SE) of 0.14 was found which is clearly below the criterion of 0.32 for an excellent measurement precision. The mean SE for the dimension variability was slightly higher than the criterion, with a value of 0.37.

**Table 6:** Standard errors per CAT dimension (N = 166).

	CAT severity	CAT impact	CAT variability
Mean SE	0.14	0.14	0.37
Standard deviation	0.08	0.09	0.06
Minimum	0.06	0.06	0.28
Maximum	0.43	0.47	0.61
Percentiles			
25	0.09	0.09	0.34
50	0.12	0.11	0.35
75	0.16	0.15	0.38

On the CAT dimension severity, 94% of the 166 cases had a SE beneath the criterion of 0.32. On the dimension impact, 93% of the cases had a SE beneath the criterion of 0.32, and on the dimension variability only 7%. Nevertheless, with regard to the variability dimension, 90% of the cases had a SE beneath 0.44, which is comparable to a classical reliability of 0.81.

### Score distribution

The fatigue scores (theta-values) on the three CAT dimensions were mainly concentrated around the value of zero (S1–S3 Figs). There were no floor- or ceiling-effects. Clearly, less than 15% of the scores were located above the value of 2 or below the value of -2 (Table 2).

## Discussion

Our results showed that, overall, the multidimensional CAT Fatigue RA has good measurement characteristics. Construct validity was supported for the CAT dimensions severity and impact with most of their examined associations with the BRAF-MDQ, BRAF-NRS and SF-36 achieving results as expected. The construct validity of the CAT dimension variability was less convincing. This result is in line with our previous finding [18] that this dimension has a less ideal model fit than the other two dimensions. We included the variability dimension in the CAT Fatigue RA because it reflects aspects that were brought up by patients [3–6] and it was part of the best fitting IRT model in our calibration study [18]. However, variability of fatigue seems to be more difficult to measure than severity and impact. The challenge is that fewer options can be asked about the variability of fatigue than about severity and impact, which complicates the development of accurate items that measure on different locations of the fatigue continuum. The Multidimensional Assessment of Fatigue [MAF] [41] is one of the few fatigue questionnaires that includes the aspect of changes in fatigue. Remarkably, a change in fatigue is covered by one single item that is not used for the calculation of the global fatigue score. To conclude, the variability dimension should not be applied in daily clinical practice or research without further examination. A logical next step is the recalibration of the CAT item bank with a two-dimensional model.

Our results reflected a very good usage of the item pool. Most of the items in the pool were selected in the 166 CAT administrations. Only some items of the impact dimension remained unused, which is not surprising since it contains about ten times more items than the severity and variability dimensions. Moreover, the CAT always administered at least five items per dimension so that a maximum of ten impact items would be administered per patient. It could be considered to leave the unused items out of the recalibration in order to streamline the item pool.

Measurement precision of the CAT was excellent for the dimensions severity and impact. Although the dimension variability had not such an excellent measurement precision, it showed a satisfactory precision in most cases.

The distribution of the fatigue scores showed no floor- and ceiling-effects. It is the characteristic of a well-functioning CAT that items are matched to the individual level of a patient. However, in order to achieve such matching, enough adequate items at the extremes of the underlying dimension have to be available from the item bank. Only minimal floor- and ceiling effects have also been found in other multidimensional CATs [24, 25]. We did not examine floor- and ceiling-effects by calculating the number of participants that responded to each item in the CAT with the highest or the lowest possible score. This is due to the fact that each patient received a different selection of twenty out of 196 possible items. In addition, the items in the item bank differ regarding the kind and number of their response options.

Four items of the SF-36 (subscale vitality) and 19 of the 20 BRAF items were part of our CAT item bank [16]. This might be a limitation of the analyses regarding construct validity, leading to superficially high correlations. However, those items only form a small part of the whole item bank (11.7%) and not all of them were selected for each CAT administration. Furthermore, we also used seven subscales of the SF-36 whose items are not part of the CAT item pool for validation purposes. In addition, the strength of the correlations found for the CAT dimensions and the other fatigue measures were comparable to those found in the evaluation study of the BRAF-MDQ [10].

Another remark concerns the BRAF-NRS coping scale. Dures et al. [32] concluded that coping with fatigue is a construct separate from severity and other dimensions, hence we expected a moderate and not a high correlation with the CAT. However, the correlations with the CAT severity and variability were low. Only the correlation with the CAT impact dimension was moderate which might be related to a small proportion of items about coping with fatigue (6.5% of 169) that are part of this dimension. Possible explanations for the low correlations are the adequate but not strong reliability of the NRS, or confusion by patients regarding high/low scores. High scores represented a positive outcome (good coping) while high scores on the NRS severity and effect indicated a negative outcome [32].

Moreover, it is difficult to compare the measurement precision of the CAT Fatigue RA with those of the BRAF-MDQ or other traditional fatigue questionnaires. For the evaluation of the standard errors on the CAT dimensions, we applied the criterion of 0.32 which corresponds to a classical reliability of Cronbachs alpha = 0.90. It is not possible to simply compare this criterion to the internal consistency of a unidimensional scale because the concepts of reliability are different. The CAT Fatigue RA is measuring in a three-dimensional space in form of an ellipse, meaning that the decline of the standard errors on the dimensions during the CAT administration can develop in a non-monotone way [38].

A recent study about the measurement of fatigue in RA showed that BRAF-MDQ, the BRAF-NRS and the subscale vitality of the SF-36 differ in their measurement precision along the fatigue continuum [42]. For example, the SF-36 is better suited to measure fatigue in patients with relatively low levels of fatigue while the BRAF-MDQ is the better choice for patients with higher levels of fatigue. Also another study [43] showed that the suitability of a measurement instrument depends on the sample in which it is used. For patients with RA and low levels of disease activity and low levels of fatigue, the items of one dimension might be most adequate. On the other hand, for patients with higher levels of fatigue, one or more additional dimensions might provide the best measurement result [43]. Our CAT contains items of different questionnaires and measures in a multidimensional way. By enabling the selection of the best suited items for the measurement of a patient’s individual fatigue level, our CAT, therefore, provides an important advantage. Furthermore, a multidimensional measurement instrument provides insight into different experiences of fatigue since it is possible to see individual compositions of scores. For example, some patients might score higher on impact, others on severity [38].

The results of this study were obtained within an outpatient sample with a relative long disease duration with a broad range. It would be interesting to test the CAT’s measurement properties in different samples of patients with RA, for example categorized by different disease durations, or by other disease characteristics.

The use of multidimensional CATs based on multidimensional IRT is a relatively recent development in health care, and clear guidelines for their design and evaluation are lacking [23]. General guidelines for the evaluation of the quality of a health-related PRO measure recommend the examination of reliability (e.g. internal consistency, test-retest reliability and measurement error), validity (content validity, criterion validity and construct validity), responsiveness and interpretability [27, 28]. Reliability of the CAT Fatigue RA was examined by means of measurement error.

Examination of other PRO measurement characteristics to evaluate the quality of our CAT Fatigue RA were considered as follows. Internal consistency refers to the correlations between different items on the same measurement instrument. For a CAT, no internal consistency in the traditional sense can be calculated because each participant receives different combinations of items. Content validity was extensively examined previously [6, 9, 15–17]. Criterion validity refers to the correlation with a gold standard [11], which is not available for the measurement of fatigue in RA [44], so that the validity of the CAT Fatigue RA was examined by means of construct validity. Interpretability was examined by means of item usage and score distribution.

Further quality standards indicate that PRO measures should be based on a conceptual and measurement model, that the patient and investigator burden is adequate and that the procedure of possible translations and their evaluation are well-documented [28]. The CAT Fatigue RA fulfils these standards. Its development started with research aiming to understand the concept of fatigue in the RA population. Consequently, items and dimensions were extensively studied and documented by incorporating expert opinions and advanced statistical methods. In fact, the concept of CAT facilitates a reduced burden for patients and investigators compared to traditional questionnaires, and the usability of the CAT Fatigue RA has been demonstrated in a previous study [30]. Translated versions are not yet available, and this is subject for future research. Finally, it has to be mentioned that the use of CAT is dependent on the availability of appropriate technical facilities and that not every patient is able or willing to fill in an online questionnaire. Consequently, using CAT in fatigue measurement might imply the risk of selection bias. However, the availability of internet access in the Netherlands is high. With a coverage ratio of internet access of 97% in general in the Netherlands and 80% in the age group of 65–75 years using internet, the reach is relatively well secured [45, 46].

This study has shown that the CAT Fatigue RA measures fatigue very precisely on different dimensions at an individual level while using a large amount of different items. The CAT Fatigue RA is a promising measurement instrument because of its unique advantage of enabling the selection of the best suited items for the measurement of a patient’s individual fatigue level along with this study’s positive findings regarding its usability [30]. Future research should investigate whether the dimension variability has to be omitted from the CAT item pool. Afterwards, its measurement properties regarding test-retest reliability, discriminative validity and responsiveness should be assessed.

## Supporting Information

S1 Data txt. (TXT)

S2 Data .sav. (SAV)

S3 Data EAP3. (EAP3)

S4 Data .dat. (DAT)

S5 Data dat. (DAT)

S6 Data .FOR. (FOR)

S7 Data .FOR. (FOR)

S8 Data TXT. Output file 1.
(TXT)

S9 Data TXT. Output file 2.
(TXT)

S1 Fig Distribution of theta values on CAT dimension “severity”. (TIF)

S2 Fig Distribution of theta values on CAT dimension „impact“. (TIF)

S3 Fig Distribution of theta values on CAT dimension „variability“. (TIF)
