# Supplementary figures and images for: Construct Validation of a Multidimensional Computerized Adaptive Test for Fatigue in Rheumatoid Arthritis
Source: PLoS One. 2015 Dec 28;10(12):e0145008. doi: 10.1371/journal.pone.0145008 (PMC4692469; doi:10.1371/journal.pone.0145008)

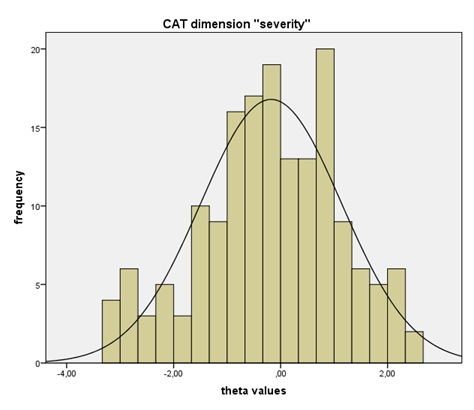

Supplement: S1 Fig — (TIF) [file pone.0145008.s010.tif]

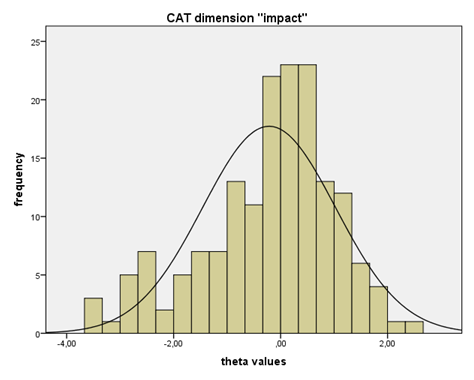

Supplement: S2 Fig — (TIF) [file pone.0145008.s011.tif]

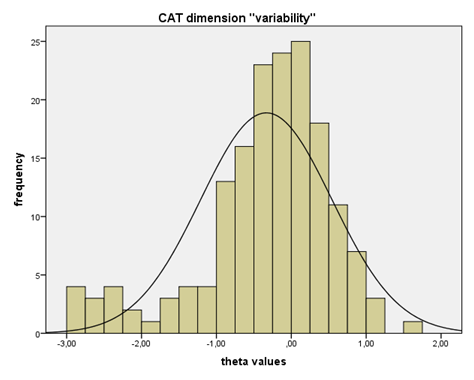

Supplement: S3 Fig — (TIF) [file pone.0145008.s012.tif]
